# The effect of encapsulated fennel extracts on the quality of silver carp fillets during refrigerated storage

**DOI:** 10.1002/fsn3.290

**Published:** 2015-12-01

**Authors:** Hoda Alipour Mazandrani, SeyedRoholla Javadian, Somayeh Bahram

**Affiliations:** ^1^ Department of FisheriesQaemshahr BranchIslamic Azad UniversityQaemshahrIran

**Keywords:** Encapsulation, fennel extract, fish preservation, liposome, silver carp

## Abstract

The effect of fennel extract on the quality of silver carp (*Hypophthalmicthys molitrix*) fillets, and the possible efficacy of liposomal encapsulation in the improvement of its antimicrobial and antioxidant activity during chilled storage (4 + 1°C) of the fillets were examined over a period of 15 days. Silver carp fillets were treated with pure fennel extract (0.3% and 0.5% w/v) and liposomal encapsulated fennel extract (0.3% and 0.5% w/v), and their quality changes in terms of total volatile basic nitrogen (TVB‐N), peroxide value (PV), thiobarbituric acid (TBA), microbial counts, and sensory properties were investigated. Fennel extract could retard the deterioration of silver carp fillets, as reflected in lower TVB‐N, PV and TBA value. Moreover, the efficacy of fennel extract was improved when it was encapsulated into liposome. Silver carp fillets treated with the encapsulated fennel extract showed the lowest amount of lipid oxidation and microbial deterioration during the storage period compared with the control and pure extract treatments. Sensory evaluation revealed that shelf life of silver carp fillet was longest for samples treated with encapsulated fennel extract at 0.5% (15 days), as compared to the control (6 days) (*P* < 0.05).

## Introduction

Fish products deteriorate rapidly as a result of high water activity, neutral pH, relatively large quantities of free amino acids, presence of autolytic enzymes, and high percent of unsaturated fatty acids (Duan et al. 2010). This problem and the increasing request for high‐quality fresh seafood has intensified the search for technologies that favor fresh fish preservation. One of the most commonly employed methods for fish preservation is cold storage. Nevertheless, it does not sufficiently prohibit the quality deterioration of fish (Jeon et al. 2002), but it can be improved using antimicrobial and antioxidant compounds.

Moreover, there is an increasing demand for natural antimicrobial and antioxidant preservatives because of the concern about safety of synthetic materials due to possible carcinogenic effects (Ozogul et al. 2010). During last decade, significant interest has been focused on natural preservatives like plant extract and essential oil as an alternative to synthetic materials. Thus, the determination of the antioxidant and antimicrobial capacity of spices and their derivate in foods is being given greater importance by researchers and those involved in the agro‐food industry (Viuda‐martos et al. 2010). Application of plant extract would not only prevent lipid oxidation and microbial deterioration of foods, but also enhance the health benefits of the foods by having the additional health‐promoting bioactivity from the herbs or spices (Ozogul et al. 2010). Among these, Fennel (*Foeniculum vulgare*) is one of the most important medicinal and aromatic plants due to its estrogenic activities and applications as a carminative, diuretic, anti‐inflammatory, antimicrobial, and galactogogue; it is a substance which is used to increase the production of milk in humans and other animals (Mahfouz 2007). Although several studies have reported the antimicrobial (Anwar et al. 2009), antifungal (Singh et al. 2006), and antioxidant (Oktay et al. 2003; Choi and Hwang 2004; Anwar et al. 2009) properties of fennel extract and essential oil, its ability in fish preservation has not been studied.

However, unfortunately, most natural active compounds are biologically unstable, poorly soluble in water, and they distribute poorly to target sites. In recent years, some novel strategies have been introduced in order to improve their stability and their bioavailability, among which is the use of liposomal encapsulation (Shoji and Nakashima 2004). Encapsulation decreases reactivity with the environment (water, oxygen, light), reduces the evaporation, or the transfer rate of the active compounds to the outside environment. It also promotes their handling ability, the bioavailability and half‐life of the compound (Fang and Bhandari 2010), masks their unpleasant taste, and increase dilution to achieve a uniform distribution in the food products when used in very small amounts (Liolios et al. 2009). Some studies (Gortzi et al. 2006) have also showed that extract encapsulation can improve antimicrobial activity of compounds and maintain the stability of antimicrobials over prolonged periods of time.

Thus, this study was aimed to investigate the effect of fennel extract on the quality of silver carp fillets, and the possible efficacy of liposomal encapsulation in the improvement of its antimicrobial and antioxidant activity during the preservation of the fillets at 4°C.

## Materials and Methods

### Materials

Liposome was obtained from Sigma‐Aldrich Chemical Co (St Louis, MO, U.S.A.). The plant fennel (*Foeniculum vulgare*) was purchased from local markets authenticated by a botanist (School of Pharmacy, Babol University of Medical Sciences, Babol, Iran). All other chemicals were analytical grade and purchased from Merck Co., Darmstadt, Germany.

### Preparation of fennel extract and liposomes

The dried fennel samples were extracted with ethanol and the extract was concentrated under vacuum (rotary evaporator). The solvent (ethanol) was added to powdered fennel in ratio of 1:10 and the resulting mixtures were shaken overnight to extract fennel's phenol compounds. After 24 h, the extracts were filtered through Whatman No. 42 filter paper to separate fennel particles. The solvents were completely evaporated in an oven at 40°C. Finally, they were placed in a refrigerator (EsmaeilzadehKenari et al. 2014).

Liposome, as carrier for fennel extract, was produced according to the method described by Gortzi et al. (2006) with some modifications. Liposome mixture was dissolved in chloroform/methanol (3/1) in a round bottom flask and the organic solvent was removed by a rotary evaporator until a thin film was formed on the walls. Fennel extract was also dissolved in dichloromethane/methanol (2/1) and mixed with liposome mixture (4/1 ratio, liposome/extract), and the solvents were evaporated under nitrogen steam. The produced lipid film was dissolved in 2 ml of phosphate buffer (10 mmol/L, pH 7) and vortexed for 15 min at 35°C. The obtained suspension was allowed to hydrate for 2 h in the dark at room temperature and then centrifuged at 6500 rpm at 4°C. Finally, multilamellar lipid vesicles were obtained by freeze‐drying.

Pure and encapsulated fennel extract was used for preparation of 0.3% and 0.5% (w/v) solution in distilled water of 4°C.

### Treatment of silver carp fillets by fennel extract

Thirty‐six live silver carp with an average weight of 1000 ± 100 g were purchased from a local aquaculture farm. In 1 h, they were transported to the laboratory in sealed foamed polystyrene boxes containing flaked ice. Then, the fishes were killed by slurry ice, skinned, filleted, and washed by potable water in a laboratory. Fifteen fillets for each treatment (100 ± 10 g fish in each group) were randomly subjected to one of the five treatments, each group individually dipped into different concentrations of fennel extracts 15 min inside a refrigerator; consequently, the dipping solution was discarded.

Five treatments as presented in the following:

C: control, without treatment
F 0.3: treatment with 0.3% pure fennel extract
F 0.5: treatment with 0.5% pure fennel extract
FE 0.3: treatment with 0.3% encapsulated fennel extract
FE 0.5: treatment with 0.5% encapsulated fennel extract

After packaging all samples in polyethylene dishes with cellophane blanket, they were stored at 4 ± 1°C for subsequent quality assessment. Chemical, microbiological, and sensory analyses were performed at 3‐day intervals to determine the overall quality of the fish for 15 days.

### Chemical analysis

#### The total volatile basic nitrogen (TVB‐N)

TVB‐N of the silver carp samples was measured by the microdiffusion method as described by Goulas and Kontominas (2005). The values were reported in mg N/100 g of fish. Measurements were repeated three times for studying repeatability.

#### Evaluation of lipid oxidation

The peroxide value (PV) was expressed in mEq oxygen/lipid and determined in the total lipid extracts according to the method of Pearson (Egan et al. 1997).

The colorimetric method described by Kirk and Sawyer (1991) was used to measure the thiobarbituric acid (TBA) value in fish fillet for secondary lipid oxidation products evaluation. All measurements were repeated three times for studying repeatability.

#### Microbiological analysis

Bacteriological counts were determined by placing a 10 g sample in 90 mL of 0.85% NaCl solution and homogenizing it with a stomacher. Total viable count (TVC) and total psychrotrophic count (TPC) was determined by the pour plate method, using plate count agar (PCA, Merk, Darmstadt, Germany). The inoculated plates were incubated at 37°C for 2 days for total viable counts, and at 10°C for 7 days for total psychrotrophic counts (Ibrahim Sallam 2007). All counts were expressed as log colony‐forming units (CFU)/g and performed in triplicate.

#### Sensory evaluation

The sensory quality of the silver carp fillets during 15 days of preservation was based on a five‐point scale. A six member trained panel was asked to judge the texture (5, firm; 1, very soft); color discoloration (5, no discoloration; 1, extreme discoloration); odor (5, extremely desirable; 1, extremely unacceptable/off‐odors); and overall acceptability (5, extremely desirable; 1, extremely unacceptable) of the samples. The fish samples were defined as unacceptable when the sensory attributes declined below 4.0 (Ojagh et al. 2010).

### Statistical analysis

The differences among all measurements were evaluated by one‐way analysis of variance (ANOVA). Duncan's multiple range tests were used to compare the means to identify which groups were significantly different from other groups. Significance was defined at *P *<* *0.05. All data are presented as mean ± SD.

## Results and Discussion

### Changes in the total volatile basic nitrogen (TVB‐N)

TVB‐N is widely studied as an indicator of deterioration of fish muscle, and measures the compounds composed of ammonia and primary, secondary, and tertiary amines (Fan et al. 2009; Abdollahi et al. 2014). According to Leroi et al. (1998), fish flesh with a level of 30 mg TVB‐N per 100 g is usually regarded as spoiled. Variations in TVB‐N values for silver carp fillets are summarized in Table 1. The initial TVB‐N value of the silver carp fillets was 11.40 mg/100 g which revealed the good quality of the fresh samples in that and freshwater fish muscle has 10–20 mg/100 g TVB‐N after harvesting (Alçiçek 2011). The value of TVB‐N increased progressively with the time of storage for all fish samples. The TVB‐N values of the samples in our study exceeded the maximum level by day 12 for control and by day 15 for samples treated with 0.3% and 0.5% fennel extract. However, TVB‐N content of the samples treated with fennel extract was significantly lower than the control during the storage period (*P* < 0.05). Lower TVB‐N content in the fillets treated with fennel extract may be related to the antibacterial activity of the extract. Antibacterial compounds like plant extracts can reduce TVB‐N production due to the decreased capacity of bacteria for oxidative deamination of nonprotein nitrogen compounds or both (Banks et al. 1980). Anwar et al. (2009) reported appreciable antimicrobial activity for fennel extract and essential oils against selected strains of bacteria and pathogenic fungi. Moreover, other authors reported lower TVB‐N content in fish fillets treated with other plant extracts like tea polyphenols (Fan et al. 2008), rosemary extract (Ozogul et al. 2010), and thyme oil (Alçiçek 2011).

**Table 1:** Changes in total volatile base nitrogen (mg N_2_/100 g) value of silver carp fillets during storage

Treatment	Storage period (days)					
	0	3	6	9	12	15
C	11.40 ± 0.12^a^	15.86 ± 0.36^a^	17.16 ± 0.45^a^	19.26 ± 0.69^a^	28.23 ± 0.06^a^	35.65 ± 0.36^b^
F 0.3	11.70 ± 0.32^a^	14.21 ± 0.16^b^	15.22 ± 0.18^b^	15.95 ± 0.46^b^	21.54 ± 0.06^b^	31.33 ± 0.26^b^
F 0.5	11.55 ± 0.22^a^	14.18 ± 0.05^b^	14.96 ± 0.34^bc^	15.84 ± 0.49^b^	21.22 ± 0.14^b^	31.06 ± 0.58^b^
EF 0.3	11.38 ± 0.34^a^	13.87 ± 0.25^bc^	14.48 ± 0.42^c^	15.41 ± 0.56^b^	19.32 ± 0.01^c^	28.42 ± 0.20^c^
EF 0.5	11.45 ± 0.15^a^	13.33 ± 0.20^c^	13.84 ± 0.24^d^	15.27 ± 0.46^b^	19.17 ± 0.06^c^	28.55 ± 0.21^c^

^a,b,c^Different small letters in the same column, represents significant difference (*P *<* *0.05). (C: control, without treatment, F 0.3: treatment with 0.3% pure fennel extract, F 0.5: treatment with 0.5% pure fennel extract, FE 0.3: treatment with 0.3% encapsulated fennel extract, FE 0.5: treatment with 0.5% encapsulated fennel extract).

In other hand, samples treated with liposomal encapsulated fennel extract showed significantly lower TVB‐N content compared to control and fillets treated with pure extract during the storage period (*P *<* *0.05). Its value remained lower than the acceptable limit for the EF 0.3 and EF 0.5 treated samples until day 15 of storage. This observation may be explained by the enhanced antimicrobial activity of the extract after encapsulation or the better protection of their functionality during the processing or storage period. Gortzi et al. (2006) also reported that after encapsulation in liposome, the antimicrobial activity of *Origanumdic tamnus* extracts proved to be higher than those of the same extracts in pure form.

### Lipid oxidation

**Figure 1:**
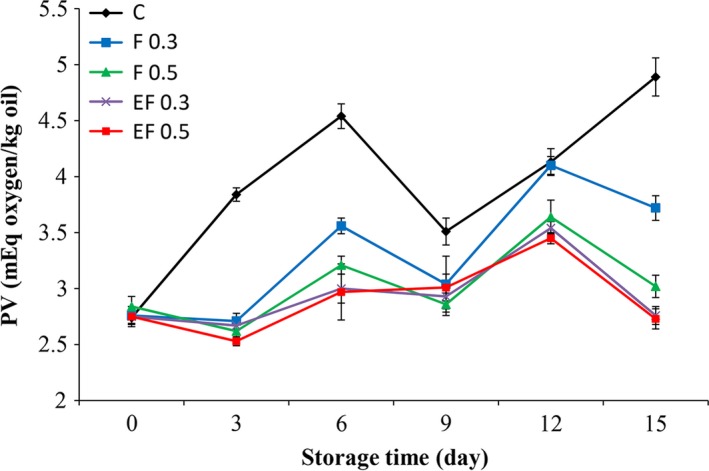
Changes in peroxide value (PV) of fish fillets during refrigerated storage. (C: control, without treatment, F 0.3: treatment with 0.3% pure fennel extract, F 0.5: treatment with 0.5% pure fennel extract, FE 0.3: treatment with 0.3% encapsulated fennel extract, FE 0.5: treatment with 0.5% encapsulated fennel extract).

The peroxide value (PV) provides a measure of the hydro peroxides which are the primary products of auto‐oxidation and they are odorless. However, their decay leads to the formation of a wide range of carbonyl compounds, hydrocarbons, furans, and other products that contribute to the rancid taste of decaying food (Yanishlieva and Marinova 2001). Variation in PV of silver carp fillets during refrigeration storage are shown in Figure 1. In this study, the PV of different treatments increased up to 6 days, after which the values fluctuated during the storage period. Nevertheless, the PV in the treated samples with pure and encapsulated extracts was significantly (*P *<* *0.05) lower than the control during the storage. These results could be attributed to the antioxidant activity of fennel extract which is related to its polyphenol contents. As explained by Turhan et al. (2009), phenolic antioxidants do not function as oxygen absorbers; rather, they inhibit the formation of fatty acid free radicals, which do react with or absorb oxygen in the auto‐oxidation process. This performance delays the onset of the auto‐oxidative process in fat or oil (Abdollahi et al. 2014). This observation was in agreement with what reported by Anwar et al. (2009) about the antioxidant properties of fennel extract. They showed that the fennel seed extracts contained appreciable levels of total phenolic contents (627.21–967.50 GAE, mg/100 g) and total flavonoid contents (374.88–681.96 CE, mg/100 g), which exhibited good inhibition of peroxidation. Our results also coincide with those of other researchers, who reported lower PV in brined anchovies (Turhan et al. 2009) and vacuum‐packed sardine (Ozogul et al. 2010) treated with myrtle and rosemary extract, respectively.

**Figure 2:**
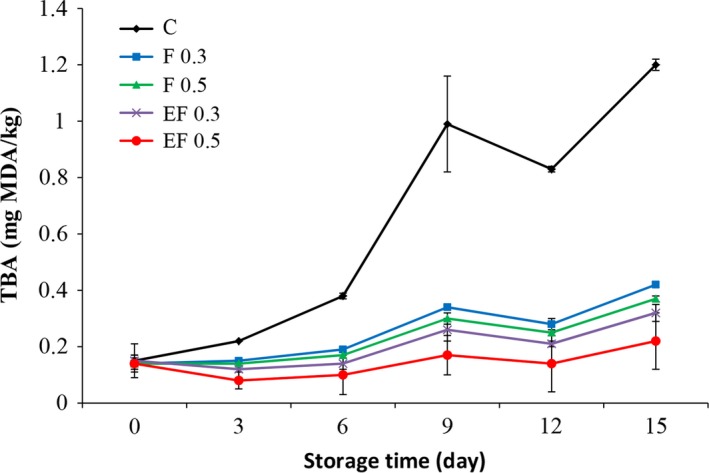
Changes in thiobarbituric acid (TBA) of fish fillets during refrigerated storage. (C: control, without treatment, F 0.3: treatment with 0.3% pure fennel extract, F 0.5: treatment with 0.5% pure fennel extract, FE 0.3: treatment with 0.3% encapsulated fennel extract, FE 0.5: treatment with 0.5% encapsulated fennel extract).

Variation in TBA values was used to describe the degree of lipid oxidation as second stage auto‐oxidation during chilled storage of silver carp fillets. Figure 2 shows the TBA values of different treatment groups during the storage period. As can be seen, the initial value of TBA was around 0.2 mg MDA/kg, closing to the value reported for silver carp by Fan et al. (2009). The TBA value of the silver carp fillets increased through the whole storage period, especially in the control samples which shows secondary lipid oxidation in the samples. However, the TBA value of the samples treated with fennel extract was significantly lower than the control during the storage, indicating that the fennel extract could be effective in reducing lipid oxidation. Other authors have also reported strong antioxidant properties for the ethanol extracts of fennel during in vitro studies (Oktay et al. 2003; Singh et al. 2006; Anwar et al. 2009), which was explained by their high phenolic content. It has been well confirmed that phenolic compounds are able to donate a hydrogen atom to the free radicals, thus, stopping the propagation chain reaction during lipid oxidation process (Singh et al. 2006). Likely, samples treated with liposomal encapsulated fennel extract showed significantly lower TBA content compared to the control and fillets treated with pure extract during the storage period (*P *<* *0.05). This may show the potential of liposomal encapsulation to improve the antioxidant activity of the fennel extract during application on the fish fillet by prolonging its availability. As mentioned before, encapsulation decreases reactivity of bioactive compound with the environment (water, oxygen, light), reduces the evaporation, or the transfer rate of the active compounds to the outside environment. It also promotes their handling ability, the bioavailability and half‐life of the compound (Fang and Bhandari 2010; Donsì et al. 2011). Evidence of liposomes improving the bioactivity and bioavailability of polyphenols has been reported by a number of researchers (Fang and Bhandari 2010). For example, Gortzi et al. (2006) reported higher antioxidant activity of *Origanum dictamnus* extracts after encapsulation in liposome.

### Changes in total viable and psychrotrophic counts

The changes in TVC with the storage period for the treated and untreated silver carp fillets are summarized in Table 2. The initial TVC of the samples was low (3.44 log10 CFU/g), indicating the high quality of fish fillets used in this study (ICMSF, 1986). Total viable count of all samples increased with storage time, and the value of control increased faster and exceeded the maximum 10^6^ log_10_ cfu/g after 6 day. This acceptability limit of 10^6^ CFU/g has been recommended for fresh fish (ICMSF, 1986). Total viable count of the fillets treated with fennel extract increased gradually and reached to 5.84, 5.80, 5.80, and 5.70 log10 CFU/g for F 0.3, F 0.5, EF 0.3, and EF 0.5, respectively, at the end of storage period. As can be seen, all treatments significantly inhibited the growth of mesophilic bacteria in silver carp compared with the control samples during the storage period. A similar trend was also observed about psychrotrophic counts in all treatments (Table 2). The lower TVC and TPC observed in samples treated with fennel extract can be related to the antibacterial activity of the extract. Shahidi Bonjar (2004) and Anwar et al. (2009) reported appreciable antimicrobial activity for fennel extract and essential oils against selected strains of bacteria and pathogenic fungi. Our results coincide with the research of Fan et al. (2008), which showed that the direct application of tea polyphenols could inhibit the growth of microorganism in silver carp fillets during refrigeration storage. Similar observations were made by Ozogul et al. (2010) about vacuum‐packed sardine treated with rosemary extract.

**Table 2:** Changes in total viable count (TVC) and total psychrotrophic count (TPC) of silver carp fillets during storage

Attributes	Treatment	Storage period (days)					
		0	3	6	9	12	15
TVC (log_10 _CFU/g)	C	3.44 ± 0.03^a^	4.72 ± 0.02^a^	6.29 ± 0.03^a^	8.34 ± 0.05^a^	8.39 ± 0.12^a^	9.82 ± 0.06^a^
	F 0.3	3.36 ± 0.07^a^	3.50 ± 0.07^bc^	3.81 ± 0.06^b^	4.07 ± 0.09^bc^	4.31 ± 0.01^b^	5.84 ± 0.11^bc^
	F0.5	3.47 ± 0.03^a^	3.53 ± 0.03^b^	3.84 ± 0.05^b^	4.11 ± 0.03^b^	4.28 ± 0.03^b^	5.80 ± 0.04^b^
	EF 0.3	3.44 ± 0.01^a^	3.50 ± 0.01^bc^	3.82 ± 0.05^bc^	4.08 ± 0.02^bc^	4.27 ± 0.02^b^	5.80 ± 0.03^bc^
	EF0.5	3.44 ± 0.08^a^	3.42 ± 0.08^c^	3.73 ± 0.01^c^	3.99 ± 0.07^c^	4.18 ± 0.07^c^	5.70 ± 0.10^c^
TPC (log_10 _CFU/g)	C	3.13 ± 0.03^a^	3.81 ± 0.14^a^	6.47 ± 0.04^a^	7.87 ± 0. 11^a^	8.14 ± 0.12^b^	8.82 ± 0.06^a^
	F 0.3	3.13 ± 0.08^a^	3.02 ± 0.08^b^	3.13 ± 0.09^bc^	3.38 ± 0.09^b^	3.48 ± 0.01^bc^	4.81 ± 0.11^bc^
	F0.5	3.11 ± 0.08^a^	3.00 ± 0.05^b^	3.11 ± 0.05^bc^	3.36 ± 0.05^b^	3.46 ± 0.03^bc^	4.78 ± 0.04^bc^
	EF 0.3	3.16 ± 0.05^a^	3.04 ± 0.05^bc^	3.17 ± 0.05^bc^	3.42 ± 0.05^b^	3.51 ± 0.02^b^	4.84 ± 0.03^b^
	EF0.5	3.08 ± 0.05^a^	2.93 ± 0.03^c^	3.04 ± 0.03^c^	3.28 ± 0.03^b^	3.38 ± 0.07^c^	4.68 ± 0.10^c^

^a,b,c^Different small letters in the same column, represents significant difference (*P *<* *0.05). (C: control, without treatment, F 0.3: treatment with 0.3% pure fennel extract, F 0.5: treatment with 0.5% pure fennel extract, FE 0.3: treatment with 0.3% encapsulated fennel extract, FE 0.5: treatment with 0.5% encapsulated fennel extract).

Furthermore, in this study, the lowest TVC and TPC were observed in the samples treated with encapsulated fennel extract. The improvement of the antimicrobial activity of natural plant extracts and essential oils when encapsulated into liposomal delivery systems was also reported by others (Gortzi et al. 2006, 2006; Liolios et al. 2009; Donsì et al. 2011). The encapsulation of eugenol and carvacrol into nanometric surfactant micelles also resulted in improved antimicrobial activity (Gaysinsky et al. 2005).

### Sensory evaluation

The results of the sensory evaluation of samples are given in Table 3. Initially, all samples had a bright and acceptable appearance. Tang was not reported by any of the panelists. Therefore, the fennel extract and encapsulation of it does not negatively affect the sensory properties of fish products. With the storage period, all sensory attributes showed a declining trend. The fish samples were considered to be acceptable for human consumption until the sensory score reached 4 (Fan et al. 2008). According to the results of the sensory analysis, acceptability of control samples were given ‘unacceptable’ scores by the 6th day, 9th day for samples treated with F 0.3%, 12th day for samples treated with F 0.5%, FE 0.3%, and 15th day for samples treated with the FE 0.5%. As storage time was extended, the samples gradually showed characteristics of strong foul order, poor texture, yellow surface color, and bad taste characteristics that are associated with spoilage of fish products due to the release of metabolites by bacteria, enzymatic action, nutrient losses, and loss of muscle flexibility (Yang et al. 2015). Silver carp treated with liposomal encapsulated fennel extract slightly higher in sensorial attributes than samples treated with pure extract which were in accordance observed for chemical properties related to antioxidant and antibacterial properties of the extracts.

**Table 3:** Changes in the sensory attributes of silver carp fillets during storage time (day)

Sensory attributes	Treatment	Storage period (days)					
		0	3	6	9	12	15
Texture	C	5.00 ± 0.00^a^	4.53 ± 0.21^b^	4.23 ± 0.21^b^	3.24 ± 0.00^c^	2.50 ± 0.00^d^	2 ± 0.04^d^
	F 0.3%	5.00 ± 0.00^a^	4.86 ± 0.20^a^	4.56 ± 0.20^ab^	4.01 ± 0.15^b^	3.78 ± 0.17^c^	3.03 ± 0.17^c^
	F 0.5%	5.00 ± 0.00^a^	4.9 ± 0.25^a^	4.75 ± 0.35^a^	4.23 ± 0.17^b^	4.12 ± 0.13^b^	3.42 ± 0.18^b^
	FE 0.3%	5.00 ± 0.00^a^	4.9 ± 0.15^a^	4.75 ± 0.25^ab^	4.13 ± 0.15^b^	4.09 ± 0.10^b^	3.34 ± 0.28^b^
	FE 0.5%	5.00 ± 0.00^a^	4.95 ± 0.15^a^	4.95 ± 0.15^a^	4.65 ± 0.15^a^	4.49 ± 0.22^a^	4.15 ± 0.15^a^
Odor	C	5.00 ± 0.00^a^	4.42 ± 0.15^a^	4.12 ± 0.15^a^	3.55 ± 0.25^b^	2.40 ± 0.23^d^	1.85 ± 0.00^d^
	F 0.3%	4.85 ± 0.15^b^	4.45 ± 0.20^a^	4.25 ± 0.20^a^	4.16 ± 0.28^a^	3.85 ± 0.27^c^	3.23 ± 0.07^c^
	F 0.5%	4.75 ± 0.3^b^	4.58 ± 0.25^a^	4.48 ± 0.15^a^	4.23 ± 0.17^a^	4.06 ± 0.03^b^	3.92 ± 0.12^b^
	FE 0.3%	4.7 ± 0.09^b^	4.53 ± 0.28^a^	4.53 ± 0.23^a^	4.23 ± 0.18^a^	4.09 ± 0.10^b^	3.72 ± 0.22^b^
	FE 0.5%	4.65 ± 0.32^b^	4.55 ± 0.25^a^	4.4 ± 0.05^a^	4.46 ± 0.25^a^	4.26 ± 0.12^a^	4.1 ± 0.05^a^
Color	C	5.00 ± 0.00^a^	4.5 ± 0.3^b^	4.1 ± 0.21^b^	3.24 ± 0.10^c^	2.15 ± 0.17^d^	1.75 ± 0.00^d^
	F 0.3%	5.00 ± 0.00^a^	4.58 ± 0.20^ab^	4.38 ± 0.20^b^	4.11 ± 0.12^b^	3.53 ± 0.37^c^	3.25 ± 0.21^c^
	F 0.5%	5.00 ± 0.00^a^	4.75 ± 0.15^a^	4.65 ± 0.25^a^	4.53 ± 0.27^a^	4.0 ± 0.05^b^	3.59 ± 0.33^bc^
	FE 0.3%	5.00 ± 0.00^a^	4.71 ± 0.05^a^	4.6 ± 0.15^a^	4.43 ± 0.25^a^	4.2 ± 0.08^b^	3.92 ± 0.22^b^
	FE 0.5%	5.00 ± 0.00^a^	4.9 ± 0.15^a^	4.85 ± 0.05^a^	4.67 ± 0.21^a^	4.52 ± 0.12^a^	4.12 ± 0.09^a^
Overall	C	5.00 ± 0.00^a^	4.55 ± 0.32^a^	4.25 ± 0.12^b^	3.33 ± 0.21^d^	2.35 ± 0.23^d^	1.38 ± 0.32^d^
	F 0.3%	4.85 ± 0.05^b^	4.75 ± 0.4^a^	4.55 ± 0.2^a^	4.00 ± 0.11^ab^	3.46 ± 0.13^c^	2.93 ± 0.08^b^
	F 0.5%	4.8 ± 0.10^b^	4.78 ± 0.09^a^	4.65 ± 0.19^a^	4.45 ± 0.07^a^	4.06 ± 0.03^b^	3.57 ± 0.02^b^
	FE 0.3%	4.75 ± 0.12^b^	4.7 ± 0.15^a^	4.65 ± 0.15^a^	4.33 ± 0.15^b^	4.05 ± 0.07^b^	3.52 ± 0.23^c^
	FE 0.5%	4.65 ± 0.15^b^	4.55 ± 0.25^a^	4.45 ± 0.15^a^	4.36 ± 0.15^a^	4.26 ± 0.12^a^	4.1 ± 0.05^a^

^a,b,c^With different small letters in the same row, represents significant difference (*P *<* *0.05). (C: control, without treatment, F 0.3: treatment with 0.3% pure fennel extract, F 0.5: treatment with 0.5% pure fennel extract, FE 0.3: treatment with 0.3% encapsulated fennel extract, FE 0.5: treatment with 0.5% encapsulated fennel extract).

## Conclusions

The effect of fennel extract on the quality of silver carp fillets and the possible efficacy of liposomal encapsulation in the improvement of its antimicrobial and antioxidant activity during chilled storage of the fillets were studied. Silver carp fillets treated with the encapsulated fennel extract showed the lowest amount of lipid oxidation and microbial deterioration during the storage period without any significant loss of texture, odor, color, or overall acceptability, while control samples had a shelf life of only 6 days. The increased efficacy of plant extract, after the encapsulation in liposomes, may promote the use of the above mentioned natural products as potent preservative in fish preservation. However, more studies are still required to optimize the encapsulation process and support the observed results for other plant extracts and essential oils.

## Conflict of Interest

None declared.
